# Mental stress and the risks of metabolic syndrome and related components in military personnel: CHIEF cohort study

**DOI:** 10.3389/fcvm.2025.1432464

**Published:** 2025-08-21

**Authors:** Kun-Zhe Tsai, Ko-Huan Lin, Ai-Hsiu Hung, Yun-Chen Chang, Xuemei Sui, Carl J. Lavie, Gen-Min Lin

**Affiliations:** ^1^ Department of Medicine, Hualien Armed Forces General Hospital, Hualien City, Taiwan; ^2^ Department of Stomatology of Periodontology, Mackay Memorial Hospital, Taipei, Taiwan; ^3^ Departments of Dentistry, Tri-Service General Hospital, National Defense Medical Center, Taipei, Taiwan; ^4^ Department of Psychiatry, Hualien Tzu Chi General Hospital, Hualien City, Taiwan; ^5^ Department of Nursing, Hualien Armed Forces General Hospital, Hualien, Taiwan; ^6^ Nursing Department, China Medical University Hospital, Taichung, Taiwan; ^7^ School of Nursing and Graduate Institute of Nursing, China Medical University, Taichung, Taiwan; ^8^ Arnold School of Public Health, University of South Carolina, Columbia, SC, United States; ^9^ John Ochsner Heart and Vascular Institute, Ochsner Clinical School, The University of Queensland School of Medicine, New Orleans, LA, United States; ^10^ Departments of Medicine, Tri-Service General Hospital, National Defense Medical Center, Taipei, Taiwan

**Keywords:** cohort study, mental stress, metabolic syndrome, military personnel, hypertension

## Abstract

**Background:**

The association observed between mental stress and metabolic syndrome (MetS) has varied across studies and may be confounded by physical activity (PA) and fitness status.

**Method:**

This study included a military cohort of 2,854 participants in Taiwan who were not taking any medications and were free of baseline MetS. The Brief Symptoms Rating Scale (BSRS-5) includes five domains—depression, anxiety, hostility, insomnia, and interpersonal sensitivity—measured on a five-point Likert-type scale ranging from 0 to 4, with a maximum score of 20. PA (hrs/wk) was categorized into three levels: <150, 150–299 and ≥300. Aerobic fitness was evaluated by the amount of time taken to complete a 3,000 m run. MetS was defined according to the International Diabetes Federation (IDF) criteria. Multivariable Cox proportional hazards regression analysis with adjustments for potential covariates including PA and aerobic fitness was utilized to determine the associations of BSRS-5 scores (each 1-unit score increase) with the incidence of MetS and related features.

**Results:**

During a median follow-up period of 5.8 years, 662 new-onset cases of MetS (23.2%) developed. BSRS-5 scores were not associated with the risk of new-onset MetS [hazard ratio (HR): 1.006 [95% confidence interval (CI): 0.975, 1.039]]. Among the five MetS features, the only one associated with BSRS-5 scores was the risk of new-onset hypertension [HR: 1.038 (95% CI: 1.002, 1.075)], which was defined as blood pressure ≥130/85 mmHg or the use of antihypertensive medications, among 2,405 participants free of baseline hypertension.

**Conclusions:**

Our findings suggest that in young adult military personnel, mental stress was not associated with the incidence of MetS but was associated with its hypertension component, which was independent of PA and aerobic fitness.

## Introduction

Metabolic syndrome (MetS), a cluster of conditions that occur together rather than a single ailment, is characterized by abdominal adiposity, hypertension, low high-density lipoprotein cholesterol (HDL-C), increased triglycerides, and insulin resistance (1, 2). People with metabolic abnormalities, whether alone or in synergy with other risk factors, face an increased risk of cardiovascular disease, diabetes, certain cancers, and an increased probability of premature death (3, 4). MetS and related conditions present substantial global public health challenges. Over a quarter of the world's population have MetS (approximately one billion people), and one-third of the Chinese population is affected (4). Recent reports also revealed that more than 50% of military personnel in the armed forces of the US and Taiwan had overweight/obesity (5, 6). Early detection of related risk factors and timely intervention are essential to prevent MetS from occurring or progressing into chronic diseases (7).

Psychological distress is also a significant public health issue in contemporary society (8). Approximately one out of eight individuals worldwide has a mental health issue, and nearly half of all people experience such issues at some point in their lives (9). Mental disorders can trigger physiological responses involving the neurological, endocrine, and immune systems, potentially contributing to MetS (4, 8, 10). For instance, eating in response to stress may result in overweight and obesity. The effect of emotional eating may be stronger among populations in high stress professions (4). Military personnel are obliged to perform regular training to maintain good fitness, which may reduce metabolic abnormalities (11). However, factors such as exposure to combat or rescue operations; witnessing traumatic events; and the demanding nature of military culture, e.g., extended deployments and a strong sense of duty, have tremendous effects on mental health which may cause emotional eating to promote overweight or obesity (12). There were only a few studies for military personnel in this context (4, 11, 13). In a recent statistics report in the U.K. army, the prevalence of overweight/obesity is over 64% in 2022–2023 (13). In the Millennium Cohort Study for U.S. military personnel, Boyko et al. have observed that mental stress, e.g., depression and anxiety was related to trouble sleep and sleep apnea that were associated with incident diabetes (14). We found that physical activity (PA) and aerobic fitness, which are crucial confounders in this association (15), were not controlled at baseline in these studies. In addition, our prior study showed an association of incident MetS with substance use which was highly related to mental stress in military personnel (16). Therefore, this study aimed to examine the association of mental stress with new-onset MetS in military personnel, with adjustments for baseline PA and aerobic fitness.

## Materials and methods

### Study participants

The cardiorespiratory fitness and health in the Eastern armed forces (CHIEF) (2) study was registered in 2014 and followed for the MetS events throughout 2020. At baseline in 2014, no participants took any antihypertensive, antidiabetic or lipid-lowering agents, and all of them received annual health examinations at the Hualien Armed Forces General Hospital, which is the only military referral hospital in Eastern Taiwan. The baseline (2014) and subsequent annual health examinations (2015–2020) consisted of subjective demographic reports and objective hemodynamic and blood metabolic biomarker measurements. In addition, participants were required to complete a questionnaire concerning their alcohol, betel nut, and tobacco use status (active vs. former/never) and PA levels at baseline (2014). Aerobic fitness was assessed by performance in a 3,000 m run test at baseline. The CHIEF study aimed to clarify the correlations and associations of PA and aerobic fitness with cardiometabolic risk in military personnel. The details of the CHIEF study have been described previously (15–22). The study protocol was approved by the Research Ethics Committee of the Mennonite Christian Hospital in Hualien City, Taiwan (certificate No. 16-05-008), and all participants provided written consent in accordance with ethical standards. Our study adhered strictly to the ethical guidelines outlined in the Declaration of Helsinki, ensuring compliance with all applicable regulations throughout the research process.

### Annual health examinations

The anthropometric waist circumference, body height and body weight of each participant were measured while the participants were standing. Body mass index (BMI) was calculated as the body weight in kilograms divided by the square of the body height in meters. The blood pressure (BP) of each participant was measured once in a seated position utilizing an automatic device (FT201 Parama-Tech Co., Ltd., Fukuoka, Japan) (23–25) after the participant had rested for 15 min or more. Venous blood samples were drawn from each participant following a 12-hour overnight fast to assess the serum levels of total cholesterol, low-density lipoprotein cholesterol (LDL-C), HDL-C, triglycerides, and fasting glucose utilizing an automated analyzer (Olympus AU640, Kobe, Japan) (26, 27).

### PA and aerobic fitness assessments

PA levels were assessed by self-reported engagement in leisure-time running over the past six months, categorized into <150, 150–299, and ≥300 min per week according to the American guidelines for exercise (12). Aerobic fitness was evaluated by time taken to complete a 3,000 m run test at the Military Physical Training and Testing Center in Hualien City, Taiwan. The 3,000 m run test was performed outdoors and conducted uniformly at 16:00 under suitable weather conditions, for example, with no heavy rain or high outdoor temperatures, e.g., >40 Celsius degrees, in accordance with military testing regulations (28).

### Mental stress assessment

Mental stress was assessed using scores on the 5-item Brief Symptom Rating Scale (BSRS-5) (6). This scale assesses mood symptoms, including trouble falling asleep (insomnia), feeling tense (anxiety), feeling easily annoyed or irritated (hostility), being in a low mood (depression), and feeling inferior to others (interpersonal sensitivity). Each domain was scored on a five-point Likert-type scale ranging from 0 (not at all) to 4 (extremely severe). The maximum BSRS-5 score is 20. Participants had to self-report BSRS-5 scores and related domain scores to assess mental stress over the past 3 months at the baseline examination. In a sample of 1,222 Taiwanese military personnel in a prior study (29), the BSRS-5 score and an additional question for suicidal ideation [on a scale from 0 (none) to 3 (severe)] were validated by the Adult Self-Report Scale-4, a self-reported rating scale including 136 items about a wide range of psychiatric symptoms of DSM-IV diagnoses (30); where the results showed moderate to high correlations regarding suicidality [*r* = 0.534–0.902].

### Definition of incident MetS events

In accordance with the International Diabetes Federation criteria specifically for Chinese individuals (1), MetS was defined as the presence of three or more of the following clinical features: (1) waist circumference ≥80 cm in women and ≥90 cm in men; (2) SBP ≥130 mmHg, and/or DBP ≥85 mmHg, or a use of anti-hypertensive medications; (3) HDL-C <50 mg/dl in women and <40 mg/dl in men; (4) triglycerides ≥150 mg/dl, or a used of lipid-lowering medications; and (5) fasting glucose ≥100 mg/dl, or a use of antidiabetic medications.

### Statistical analysis

The baseline characteristics of the study cohort are presented as the mean ± standard deviations (SD) for continuous variables and as number (percentage) for categorical variables. Follow-up for each participant began at baseline (2014) and continued until the first occurrence of MetS, loss to follow-up, or the end of the follow-up period (December 31, 2020). The severity of mental stress was classified into three categories based on BSRS-5 scores ≤1, 2, and ≥3 or treated as a continuous variable (each 1-unit increase). We utilized multivariable Cox proportional hazards regression models to investigate the association between BSRS-5 score and incident MetS. Model 1 is crude model. In Model 2, age, sex, waist circumference, SBP, DBP, HDL-C, triglycerides, and fasting glucose are adjusted at baseline. Model 3 includes adjustments for substance use status and the covariates in Model 2. Model 4 includes adjustments for baseline PA levels, aerobic fitness, and the covariates in Model 3. All confounders were chosen for an association with mental stress or MetS in priorly published reports (18, 23, 25). As is known, Cox regression model is the most commonly used method for the analysis of survival data, particularly in some well-known observational cohort studies (11, 31) since it does not require the assumption of survival distributions for the data (32). Statistical significance was defined as two-sided *P* values <0.05. All statistical analyses were performed using SPSS v26.0 software for Windows (IBM Corp., Armonk, NY, USA).

## Results

**Figure 1:**
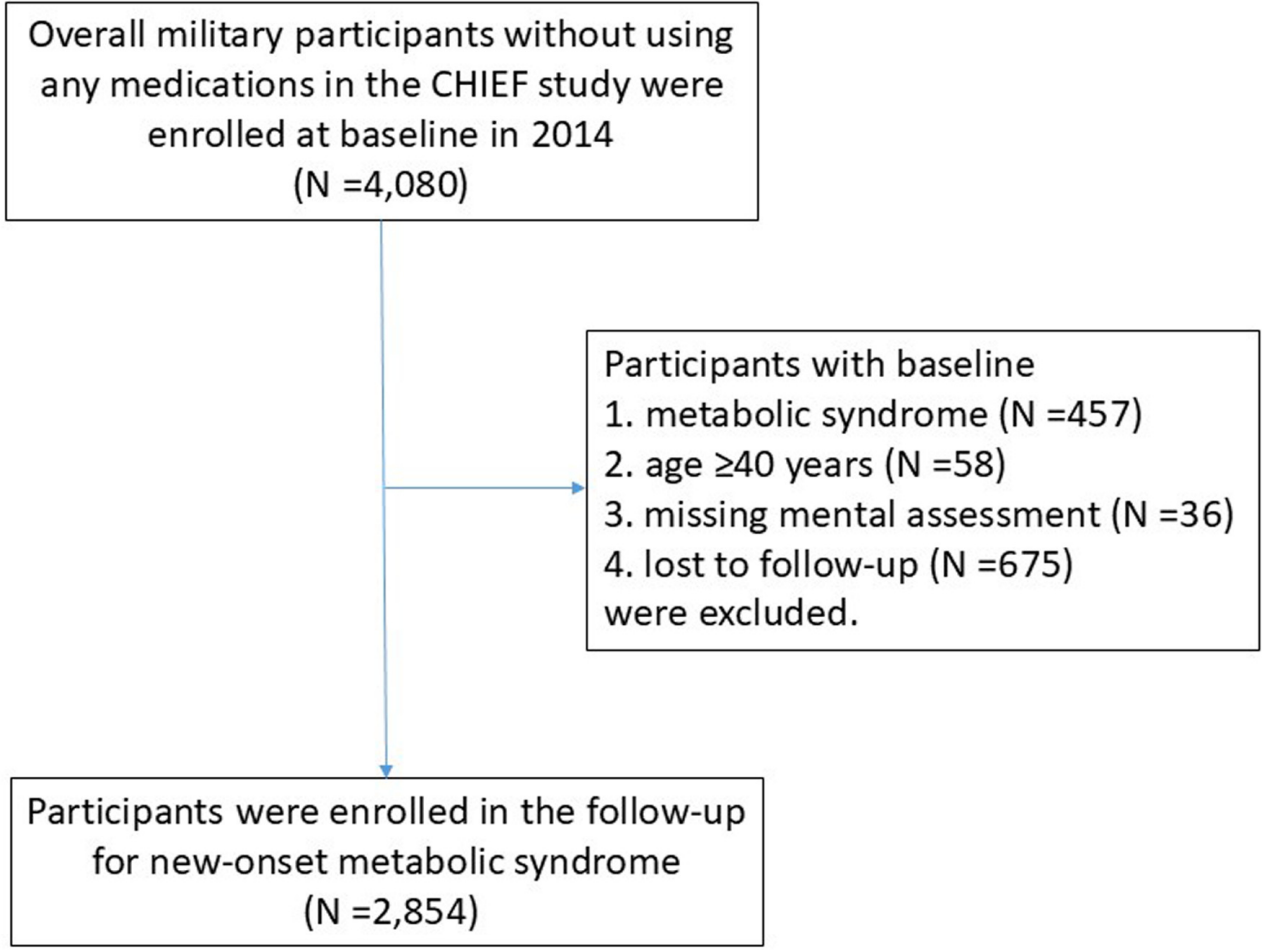
The flowchart to select eligible participants at baseline (2014) for the follow-up of new-onset metabolic syndrome in the CHIEF study, 2014–2020.

The CHIEF cohort study recruited 4,080 military personnel at baseline (2014). Participants meeting specific criteria were excluded at baseline, i.e., those with MetS (*N* = 457), those aged 40 years or older (*N* = 58), those with missing mental health information (*N* = 36), and those who left the military bases in Eastern Taiwan area and were lost to follow-up (*N* = 675). Ultimately, a total of 2,854 participants were included in the final analysis (Figure 1). Over a mean 6-year period of follow-up from January 1, 2014, to December 31, 2020, we identified 662 (23.2%) incident cases with new-onset MetS. Table 1 reveals the baseline demographic and clinical characteristics of the study cohort. The participants who developed MetS were older and had a larger waist circumference; higher BMI, SBP, and DBP; elevated serum total cholesterol, LDL-C, triglycerides and fasting glucose levels; and lower HDL-C levels at baseline (Table 1) than those who did not develop MetS. Additionally, the participants with new-onset MetS were observed to have a higher prevalence of active substance use, i.e., alcohol intake, betel nut chewing and cigarette smoking. Moreover, their aerobic fitness was marginally lower in PA levels (*p* = 0.09).

**Table 1:** Baseline characteristics of participants.

Variables	Total participants (*N* = 2,854)	Participants without new-onset MetS (*N* = 2,192)	Participants with new-onset MetS (*N* = 662)	*P*
BSRS-5 score	1.87 ± 2.67	1.83 ± 2.63	2.01 ± 2.80	0.12
≤1, %	1,788 (62.6)	1,384 (63.1)	404 (61.0)	0.55
2, %	213 (7.5)	164 (7.5)	49 (7.4)	
≥3, %	853 (29.9)	644 (29.4)	209 (31.6)	
Age, years	28.37 ± 5.79	27.81 ± 5.82	30.24 ± 5.28	<0.001
Male sex, %	2,545 (89.2)	1,909 (87.1)	636 (96.1)	<0.001
Waist circumferences, cm	81.43 ± 8.06	79.73 ± 7.69	87.09 ± 6.55	<0.001
BMI, kg/m^2^	24.27 ± 3.00	23.62 ± 2.83	26.41 ± 2.51	<0.001
SBP, mmHg	115.88 ± 12.90	114.53 ± 12.77	120.33 ± 12.31	<0.001
DBP, mmHg	69.29 ± 9.66	68.41 ± 9.50	72.21 ± 9.63	<0.001
Blood test				
Total cholesterol, mg/dl	171.98 ± 32.40	168.96 ± 30.66	181.97 ± 35.83	<0.001
LDL-C, mg/dl	103.77 ± 28.79	100.61 ± 27.60	114.24 ± 30.15	<0.001
HDL-C, mg/dl	49.96 ± 10.03	51.39 ± 10.03	45.25 ± 8.48	<0.001
Triglycerides, mg/dl	96.89 ± 58.67	86.95 ± 45.38	129.79 ± 81.37	<0.001
Fasting glucose, mg/dl	92.10 ± 9.75	91.19 ± 8.89	94.72 ± 11.79	<0.001
Substance use, %				
Alcohol intake, active	1,160 (40.6)	823 (37.5)	337 (50.9)	<0.001
Betel-nut chewing, active	279 (9.8)	174 (7.9)	105 (15.9)	<0.001
Tobacco smoking, active	991 (34.7)	711 (32.4)	280 (42.3)	<0.001
PA levels, %				
<150 min/week	592 (20.7)	473 (21.6)	119 (18.0)	0.09
150–299 min/week	1,104 (38.7)	848 (38.7)	256 (38.7)	
≥300 min/week	1,158 (40.6)	871 (39.7)	287 (43.4)	
Aerobic fitness, seconds	868.44 ± 81.14	866.04 ± 82.61	876.85 ± 75.25	0.005

Continuous variables are presented as mean ± standard deviation (SD), and categorical variables as *N* (%).

BSRS, brief symptom rating scale; BMI, body mass index; SBP, systolic blood pressure; DBP, diastolic blood pressure; LDL-C, low-density lipoprotein cholesterol; HDL-C, high-density lipoprotein cholesterol; PA, physical activity.

Table 2 demonstrates the results of the multivariable Cox regression analysis examining incident new-onset MetS in relation to baseline BSRS-5 scores. There was no association found in either the crude model or the multivariable models, regardless of whether the BSRS-5 score was treated as a continuous variable or a categorical variable.

**Table 2:** Association of BSRS-5 score with incident new-onset metabolic syndrome.

Mental stress grades	*N*	Events	Model 1 (Crude)	*P*-value	Model 2	*P*-value	Model 3	*P*-value	Model 4	*P*-value
			HR (95% CI)		HR (95% CI)		HR (95% CI)		HR (95% CI)	
BSRS-5 score	2,854	662	1.021 (0.991–1.052)	0.17	1.003 (0.973–1.035)	0.83	1.000 (0.969–1.032)	0.98	1.006 (0.975–1.039)	0.70
≤1	1,788	404	1.000		1.000		1.000		1.000	
2	213	49	0.990 (0.719–1.364)	0.95	0.908 (0.658–1.254)	0.55	0.894 (0.647–1.236)	0.49	0.924 (0.667–1.278)	0.63
≥3	853	209	1.051 (0.876–1.260)	0.59	0.986 (0.818–1.189)	0.88	0.972 (0.804–1.176)	0.77	1.011 (0.830–1.231)	0.91
*P* for trend				0.60		0.83		0.72		0.95

Data are shown as hazard ratio (HR) and 95% confidence interval (CI) with adjustments for baseline age, sex, waist circumferences, systolic blood pressure, diastolic blood pressure, high-density lipoprotein cholesterol, triglycerides and fasting glucose in Model 2, and additional adjustments for substance use status in Model 3, and additional adjustment for physical activity levels and aerobic fitness in Model 4.

BSRS, brief symptom rating scale.

Table 3 shows the results for the incidences of various metabolic abnormalities in relation to BSRS-5 scores. When BSRS-5 scores were treated as a continuous variable, higher scores were associated with a greater incidence of new-onset hypertension [hazard ratio (HR): 1.038 (95% confidence interval (CI): 1.002, 1.075)]. Similarly, when BSRS-5 scores were treated as a categorical variable, a tendency toward a higher BSRS-5 score (2 and ≥3) was associated with a greater risk of new-onset hypertension than the BSRS-5 scores ≤1 [HRs: 1.131 (95% CI: 0.786, 1.627) and 1.351 (95% CI: 1.081, 1.689), respectively; *p* for trend = 0.008]. However, the other MetS clinical features (abdominal obesity, prediabetes, low HDL-C, and elevated triglycerides) showed no associations with BSRS-5 scores.

**Table 3:** Association between BSRS-5 score and incidence of each metabolic syndrome feature.

Mental stress grades	*N*	Events	Model 1 (Crude)	*P*	Model 2	*P*	Model 3	*P*	Model 4	*P*
			HR (95% CI)		HR (95% CI)		HR (95% CI)		HR (95% CI)	
Central obesity										
BSRS-5 (cont.)	2,385	537	1.021 (0.988–1.055)	0.21	1.006 (0.974–1.040)	0.70	1.007 (0.974–1.041)	0.68	1.010 (0.977–1.045)	0.54
BSRS-5 ≤ 1 (ref.)	1,489	342	1.000		1.000		1.000		1.000	
2	163	36	0.871 (0.598–1.269)	0.47	0.980 (0.668–1.438)	0.91	0.974 (0.663–1.430)	0.89	0.993 (0.675–1.460)	0.97
≥ 3	706	159	1.021 (0.837–1.247)	0.83	1.017 (0.831–1.245)	0.86	1.022 (0.832–1.256)	0.83	1.050 (0.851–1.296)	0.65
*P* for trend				0.89		0.87		0.84		0.66
Hypertension										
BSRS-5 (cont.)	2,405	466	1.027 (0.993–1.061)	0.12	1.025 (0.990–1.060)	0.16	1.029 (0.994–1.065)	0.10	1.038 (1.002–1.075)	0.03
BSRS-5 ≤ 1 (ref.)	1,492	268	1.000		1.000		1.000		1.000	
2	185	40	1.127 (0.787–1.614)	0.51	1.063 (0.741–1.524)	0.74	1.077 (0.750–1.546)	0.68	1.131 (0.786–1.627)	0.50
≥ 3	728	158	1.174 (0.951–1.449)	0.13	1.230 (0.994–1.552)	0.06	1.271 (1.023–1.578)	0.03	1.351 (1.081–1.689)	0.008
*P* for trend				0.12		0.06		0.03		0.008
Low HDL-C										
BSRS-5 (cont.)	2,427	372	1.036 (0.998–1.076)	0.062	1.015 (0.977–1.055)	0.43	1.016 (0.978–1.057)	0.41	1.021 (0.981–1.062)	0.31
BSRS-5 ≤ 1 (ref.)	1,523	226	1.000		1.000		1.000		1.000	
2	184	31	1.145 (0.771–1.701)	0.50	1.132 (0.760–1.685)	0.54	1.114 (0.747–1.660)	0.59	1.131 (0.757–1.689)	0.54
≥ 3	720	115	1.106 (0.873–1.402)	0.40	0.999 (0.784–1.273)	0.99	1.015 (0.793–1.299)	0.90	1.042 (0.806–1.347)	0.75
*P* for trend				0.37		0.96		0.86		0.71
Elevated triglycerides										
BSRS-5 (cont.)	2,521	543	1.007 (0.974–1.041)	0.68	1.004 (0.970–1.039)	0.81	0.993 (0.959–1.029)	0.70	0.997 (0.962–1.034)	0.88
BSRS-5 ≤ 1 (ref.)	1,583	346	1.000		1.000		1.000		1.000	
2	189	39	0.806 (0.560–1.160)	0.24	0.669 (0.485–1.008)	0.055	0.678 (0.470–0.978)	0.03	0.695 (0.481–1.004)	0.052
3	749	158	0.955 (0.783–1.165)	0.65	0.956 (0.782–1.169)	0.66	0.902 (0.735–1.108)	0.32	0.926 (0.750–1.143)	0.47
*P* for trend				0.56		0.51		0.23		0.36
Elevated fasting glucose										
BSRS-5 (cont.)	2,496	495	1.018 (0.984–1.053)	0.30	1.012 (0.978–1.049)	0.48	1.012 (0.976–1.049)	0.52	1.013 (0.977–1.051)	0.48
BSRS-5 ≤ 1 (ref.)	1,558	292	1.000		1.000		1.000		1.000	
2	186	33	0.922 (0.628–1.353)	0.67	0.918 (0.625–1.349)	0.66	0.915 (0.622–1.345)	0.65	0.926 (0.629–1.364)	0.69
≥ 3	752	170	1.176 (0.962–1.439)	0.11	1.139 (0.929–1.396)	0.21	1.144 (0.927–1.413)	0.21	1.167 (0.938–1.451)	0.16
*P* for trend				0.13		0.23		0.23		0.18

Data are shown as hazard ratio (HR) and 95% confidence interval (CI) with adjustments for baseline age, sex, waist circumferences, systolic blood pressure, diastolic blood pressure, high-density lipoprotein cholesterol, triglycerides and fasting glucose in Model 2, and additional adjustment for substance use status in Model 3, and additional adjustment for physical activity levels and aerobic fitness in Model 4.

BSRS, brief symptom rating scale; cont., continuous variable; HDL-C, high density lipoprotein cholesterol; ref., reference.

## Discussion

To the best of our knowledge, this study represents the first investigation into the associations between mental stress and the incidences of new-onset MetS and related metabolic components in a young military population. We did not identify an association between mental stress assessed by the BSRS-5 score and incident new-onset MetS, despite controlling for various potential confounders, including baseline PA levels, aerobic fitness, and other covariates. Nevertheless, our findings suggest a dose‒response association between mental stress grades and new-onset hypertension.

Based on the complex pathomechanisms of stress, a correlation between mental stress and MetS appears to be justified. Mental stress activates the sympathetic nervous system and adrenal medulla, resulting in catecholamine release, and triggers the hypothalamic‒pituitary‒adrenal (HPA) axis which increases blood cortisol levels (31). Both catecholamines and cortisol contribute to elevated blood glucose levels and insulin resistance, which are key factors in MetS. Mental stress also induces the chemokines and inflammatory cytokines release which promote the development of insulin resistance, diabetes and MetS (8, 33, 34). The stress-induced inflammatory process may involve sympathetic nervous system activation and NF-*κ*B transcription factor activation (35). Psychiatric disorders such as bipolar disorder and posttraumatic stress disorder (3, 36–38), and mental stress (8, 39), have been found as risk factors for MetS. However, findings from a cohort study suggested that mental stress might be a consequence rather than a cause of MetS (40). Another study reported a specific association of depression with MetS in a hospital sample in Taiwan (41). Our results may differ from those of prior studies due to variations in the definition of mental stress. Furthermore, previous meta-analyses have revealed varying impacts of stress on MetS, with occupational stress having the strongest impact and general perceived stress having a weaker impact (42). Additionally, our assessment of mental stress relied on self-reports rather than objective clinical diagnoses, which might be another possible reason accounting for the null association between mental stress and incident MetS in this study. Consistent with our findings, several studies also reported no relationship between MetS and mental stress (4, 8, 11, 13, 43).

A growing body of evidence suggests an association between mental stress and hypertension (4, 40, 44–47), particularly affecting DBP instead of SBP (42, 48). In a meta-analysis by Liu et al., those with mental stress had a twofold increase in their probability of hypertension (odds ratio: 2.40, 95% CI: 1.65–3.49) (46). In contrast, a few studies showed no association between mental stress and hypertension (48, 49). A study for Ghanaians in Amsterdam demonstrated that mental stress, e.g., permitted discrimination, depression and financial stress, did not increase the prevalence of hypertension (49). Chronic exposure to mental stress could sustain this heightened sympathetic activity, which may lead to cardiovascular issues such as cardiac arrhythmias, elevated BP, and, in severe cases, sudden death (47). Chronic mental stress may affect neuroglial circuits or compromise antioxidant systems, possibly altering the response of neurons to stress (47). Other metabolic abnormalities, specifically, central obesity, insulin resistance, and dyslipidemia, have also been reported to be linked to mental stress in some prior studies (8, 39). Nevertheless, PA and aerobic fitness were rarely controlled in the prior studies to address this issue, which may lead to a bias (50). Military personnel belong to a distinct group and are commonly required to engage in rigorous PA to have superior fitness. This may counteract the negative impact of mental stress on metabolic health.

Several limitations should be considered when interpreting the data in this study. First, the self-report questionnaire used in this study to assess mental health provided only suboptimal evidence compared with clinical diagnosis. Second, military personnel represent a specific population, and the small sample size of female participants in this study may restrict the generalizability of our results to the broader public. Third, our results do not appear to be adjusted for relevant social and psychological factors. This is a significant limitation, as such factors are known to influence both mental stress and metabolic outcomes. In contrast, this study was strengthened by the detailed data on the baseline characteristics of the participants, which facilitated adjustments for potential confounding factors. Lifestyle factors such as PA and diet can affect both mental stress and the development of MetS in the general population. The uniformity among military personnel regarding their living environment, diets, and health care provisions helps minimize the impact of unmeasured variables. In addition, several crucial confounders that have been relatively understudied in prior research, such as PA and aerobic fitness levels, were controlled for at baseline.

## Conclusions

Our findings suggest that mental stress may not be effective in predicting the development of MetS, but it does increase the risk of new-onset hypertension among military populations. Based on our study findings, prevention of new-onset hypertension should be emphasized and initiated in military personnel who experience high mental stress. Future studies specifically focusing on the military population will be required. It is crucial to consider the realities of this population across different countries in order to deepen understanding of the relationships between mental stress, MetS, obesity, and other chronic diseases.

## Data Availability

The raw data supporting the conclusions of this article will be made available by the authors, without undue reservation.
